# Precision therapeutic opioid dosing implications from genetic biomarkers and craving score

**DOI:** 10.1097/MD.0000000000020429

**Published:** 2020-05-29

**Authors:** Hsin-Wen Chang, Wen-Chao Ho, Chieh-Liang Huang, Ruey-Yun Wang

**Affiliations:** a Department of Public Health, China Medical University, Taichung; b Center for General Education, Hsuan Chuang University, Hsinchu City; c Brain Disease Research Center; d Center for Drug Abuse and Addiction, China Medical University Hospital, Taichung, Taiwan; e College of Medicine, China Medical University, Taichung, Taiwan.

**Keywords:** genetic risk score, methadone maintenance treatment, prediction, quantitative trait locus

## Abstract

Determining the clinically optimal dose in methadone maintenance therapy (MMT) is a time-consuming procedure, which considers clinical signs and symptoms.

To perform a quantitative trait locus association for identifying genetic variants for MMT dosage that underlie heroin addiction and methadone metabolism and then integrate several genotypic and phenotypic factors are potential predictors for clinically optimal MMT dose for personalized prescription.

In total, 316 heroin-dependent patients undergoing MMT were recruited at the Addiction Center of the China Medical University Hospital. A multinomial logistic regression model was used to assess associations between genetic polymorphisms and MMT dosing. The data were randomly separated into training and testing sets. In order to enhance the prediction accuracy and the reliability of the prediction model, we used areas under the receiver operating characteristic curves to evaluate optimal MMT dose in both training and testing sets.

Four single nucleotide polymorphisms, namely rs806368 in *CNR1*, s1386493 in *TPH2*, s16974799 in *CYP2B6*, and rs2229205 in *OPRL1*, were significantly associated with the maximum MMT dose (*P* < .05). The genetic risk score (GRS) was associated with maximum MMT dose, and after adjustments for age, sex, and body mass index, the GRS remained independently associated with the maximum MMT dose. The area under the receiver operating characteristic curve of the combined GRS and craving score was 0.77 for maximum MMT dose, with 75% sensitivity and 60% specificity.

Integrating the GRS and craving scores may be useful in the evaluation of individual MMT dose requirements at treatment initiation. Optimal dose prediction allows clinicians to tailor MMT to each patient's needs.

## Abstract 2

Supplemental Digital Content is available in the text

## 1 Introduction

Heroin addiction, a chronic relapsing disease characterized by compulsive drug seeking, drug abuse, tolerance, and physical dependence, is a major public health concern worldwide.^[1]^ Methadone, a synthetic racemic opioid that primarily functions as a μ-opioid receptor agonist,^[2]^ is commonly used to treat heroin addiction; it reduces cravings, withdrawal symptoms, risky behavior incidence, and relapse risk.^[3]^ When prescribing methadone, the dosing requires considerable care: excessive methadone doses are dangerous, whereas overly conservative doses are ineffective at preventing relapse to heroin use.^[4]^ However, the effective methadone maintenance therapy (MMT) dose varies for every individual and is the key to safe and successful treatment for heroin addiction.^[5]^ The clinically optimal dose should be determined on the basis of clinical signs and symptoms, which is a time-consuming process. MMT remains a crucial treatment strategy for heroin addiction.

Methadone is extensively metabolized by cytochrome P450 (*CYP*) in the liver.^[6–8]^ Several studies have suggested that numerous genetic variants may play influential roles in the etiology of methadone metabolism and μ-opioid receptors.^[7,9,10]^ Furthermore, other genes of the opioid system or genes involved in serotoninergic, dopaminergic, or noradrenergic pathways have been associated with MMT.^[5]^ Polymorphisms in genes related to these pathways may contribute to interindividual variability in optimal MMT dose and have also been associated with different aspects of drug addiction.^[11]^ However, genetic factors, which may play a key role in determining the methadone dosage, are generally not evaluated in clinical practice.

To identify genetic variants for MMT dosage that underlie heroin addiction and methadone metabolism, we performed a quantitative trait locus (QTL) association study using single nucleotide polymorphisms (SNPs) selected from candidate genes. This approach is based on physiological hypotheses in which the candidate genes were selected on the basis of their function (eg, opioidergic, dopaminergic, and serotonergic, and ethadone-metabolizing) and the related pathways (eg, cognitive function). We focused on 13 genes encoding opioidergic components (*OPRK1*, *OPRL1*, *PDYN*, and cannabinoid receptor 1 [*CNR1*]), dopaminergic and serotonin components (*COMT* and *tryptophan hydroxylases 1 [*TPH1*] and 2* [*TPH2*]), methadone-metabolizing enzymes (*CYP1A2, CYP2B6,* and *CYP2C1*), as well as cognitive function–related genes (*GRIN3A*, *GRIN3B*, and *GRM6*). To maximize the power of the study, cases were selected from the extreme margin of the specific phenotype range (eg, severe heroin addicts receiving MMT). We combined several genotypic and phenotypic factors that could potentially serve as predictors of clinically optimal MMT dose for personalized analgesic prescription.

## 2 Materials and methods

### 2.1 Patients

The study protocol was reviewed and approved by the Institutional Review Board of China Medical University Hospital (CMUH) (DMR101-IRB1-218) and was in compliance with the Declaration of Helsinki. In total, 316 heroin-dependent patients undergoing MMT were recruited at the Addiction Center of the CMUH. As assessed by senior psychiatrists experienced in heroin dependence, all recruited patients met Diagnostic and Statistical Manual of Mental Disorders, 4th Edition, criteria for heroin abuse and dependence and had been receiving MMT for at least 6 months with an unchanged dose for at least 4 weeks at CMUH. Moreover, the recruited patients provided written informed consent, had normal electrocardiography results, and were not using concurrent medications that possibly affected methadone metabolism. For each patient, the following clinical information was recorded: sex, weight (in kg), height (in cm), liver function test results, comorbidities, and daily methadone dose.

### 2.2 Heroin use and craving questionnaire

We designed a 14-item heroin use and craving (HUC) questionnaire to evaluate the behavior of heroin users undergoing MMT. Each question was scored from 0 to 4. The questionnaire was divided into 2 parts: Part I (HUC 1–6, possible total scores: 0–24), assessing the urge for heroin and whether the individual can shift the attention away from heroin, and Part II (HUC 7–14, possible total scores: 0 to 32), investigating the daily or weekly frequency of heroin usage, daily life disturbance, anxiety, and ability to overcome heroin use. In both the part, the higher the score, the more severe the heroin craving was. The score of each item was calculated according to the manual.

### 2.3 Candidate variants selection and genotyping

This study focused on the 13 aforementioned genes encoding various metabolic components: *OPRK1*, *OPRL1*, *PDYN*, *CNR1*, *COMT*, *TPH1*, *TPH2*, *CYP1A2, CYP2B6*, *CYP2C1*, *GRIN3A*, *GRIN3B*, and *GRM6*. SNPs with minor allele frequency >0.05 were selected on the basis of the previous findings and population data in SNP databases of the National Center for Biotechnology Information Han Chinese in Beijing, China In total, 44 SNPs were genotyped at the National Center for Genome Medicine, Taiwan by using Sequenom iPLEX matrix-assisted laser desorption/ionization time-of-flight mass-spectrometry technology. DNA was extracted from 3 to 10 mL of whole blood by using QIAamp DNA Blood Mini Kit (Qiagen, Valencia, CA) according to the manufacturer's protocol. Duplicate samples were randomly selected for quality control and the concordance rate was >0.99 for all SNPs assayed.

We calculated an SNP risk score based on estimates of the odds ratio per allele and risk allele frequencies assuming independence of additive risks (22). A score of 1 was given to each T allele of rs806368 in *CNR1* and each C allele of rs1386493 in *TPH2*, rs16974799 in *CYP2B6*, and rs2229205 in *OPRL1*. The overall genetic risk score (GRS) was then calculated by using the SNP risk alleles.

### 2.4 Statistical analysis

Each SNP genotype frequency distribution was examined for Hardy–Weinberg equilibrium by using the chi-square 1 degree of freedom goodness-of-fit test. Demographic characteristics and clinical parameters were evaluated with a chi-squared contingency table for categorical variables and *t* test for continuous variables for both sexes. We performed QTL mapping for maximum methadone maintenance dose. The general linear model was used to associate maximum methadone maintenance dose with genotype data, making adjustments for sex, age, and body mass index (BMI). GRSs ranged from 0 to 4. A multinomial logistic regression model including GRS and craving score, adjusted for covariates, was applied to examine the association between maximum methadone maintenance dose and genetic variants. The data were randomly separated into training and testing sets. In both splits, the training and testing data generally follow similar characteristics distributions without significant differences (Supplementary Table 1, http://links.lww.com/MD/E366). In order to enhance the prediction accuracy and the reliability of the prediction model, we calculated the areas under the receiver operating characteristic curves (AUROCs) to compare the accuracy of maximum MMT dose. Sensitivity, specificity, and AUROCs were used to evaluate model performance in both training and testing sets. The optimal cutoff risk score threshold was identified at which both sensitivity and specificity were maximized (sensitivity + specificity). In addition, AUROC loss by stepwise selection was also performed to investigate critical and determining factors of the model.

## 3 Results

### 3.1 Patient characteristics

No significant differences were observed between male and female patients with respect to their HUC questionnaire score, maximum dose, heroin use duration, education level, and marital status, but significant differences were noted in the mean age, BMI, and heroin onset age (Table 1).

**Table 1:**
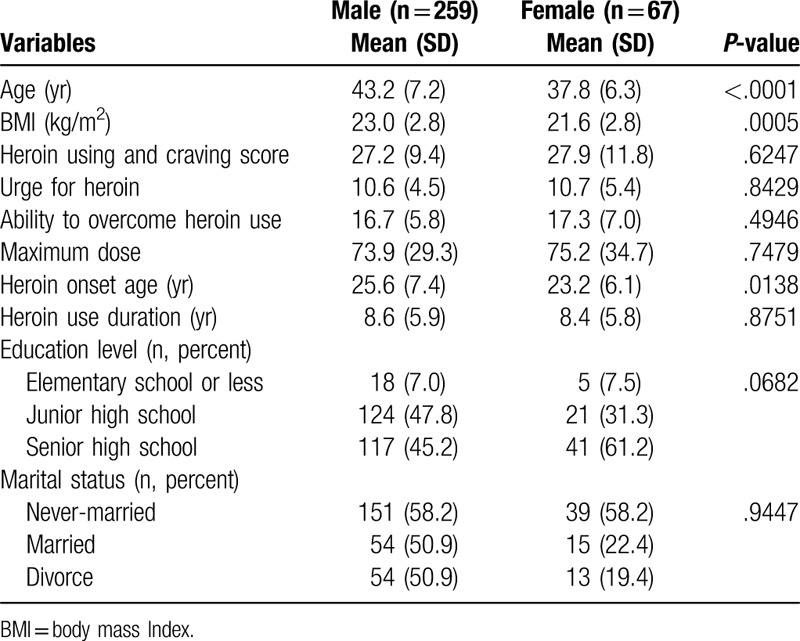
Comparing characteristics of the study subjects.

In total, 40 SNPs in 10 genes were not associated with maximum MMT dose (Supplementary Table 2, http://links.lww.com/MD/E366). Only 4 SNPs, rs806368 in *CNR1*, s1386493 in *TPH2*, s16974799 in *CYP2B6*, and rs2229205 in *OPRL1*, were significantly associated with maximum MMT dose (*P* < .05; Table 2)

**Table 2:**
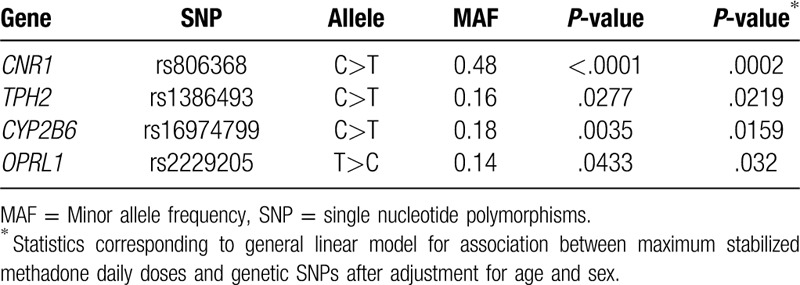
Association between selected SNPs and maximum stabilized methadone daily doses.

We calculated the GRS by using 4 significant SNPs from Table 3. The univariate associations of GRS and the associations with maximum MMT dose are presented in a dose–response manner in Table 3. The GRS was associated with maximum MMT dose. Even after adjustments for age, sex, and BMI, the GRS remained independently associated with maximum MMT dose.

**Table 3:**
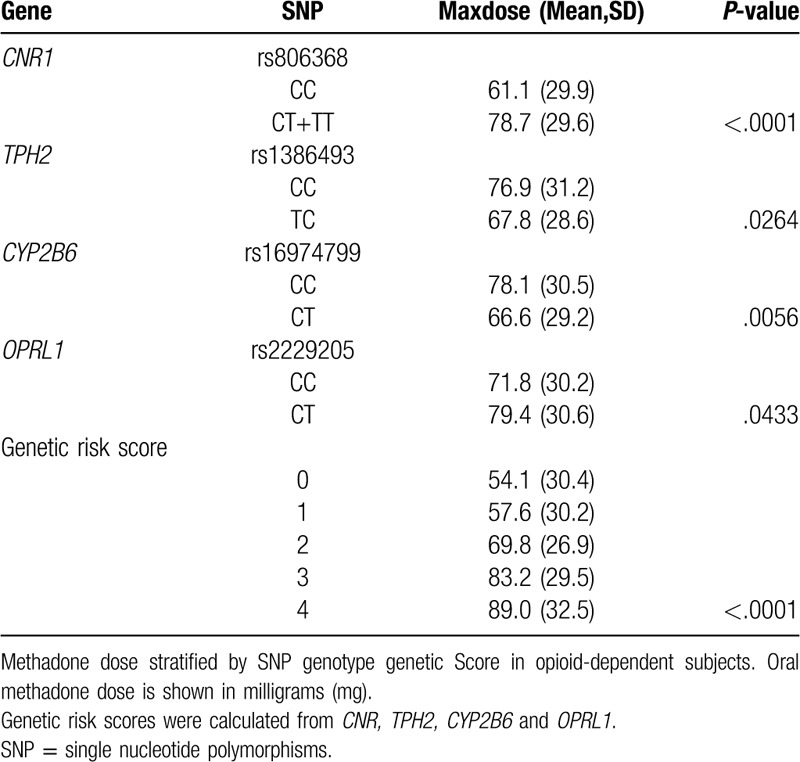
Predicted maximum stabilized methadone daily doses for genetic risk scores.

### 3.2 Selection of predictive variables for maximum MMT dose

On the basis of the results presented in Table 3, we conducted stepwise, backward and forward model analysis to select the determining factors for the best fit prediction model of maximum MMT dose. The results are displayed in Table 4. *CNR1*, *TPH2*, *CYP2B6*, *OPRL1*, and craving were included in the best fit model, with *P* set at <.05.

**Table 4:**
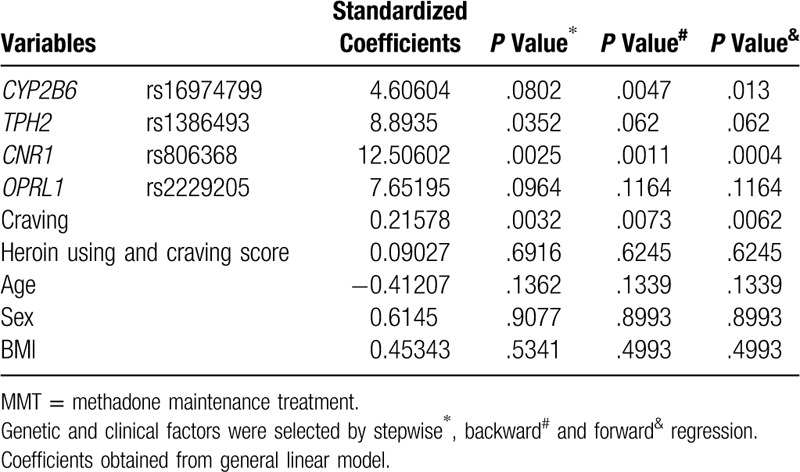
Selection of predictive variables for maximum dose of MMT.

In order to enhance the prediction accuracy and the reliability of the prediction model, the dataset was randomly split into training [229 (70%) subjects] and testing [97(30%) subjects] sets. Logistic regression model were trained and optimized using the training set and their performances was evaluated using the test set. This performance was replicated on the testing set and has a similar AUROC for both training set (AUROC = 0.75) and testing set (AUROC = 0.81) (Supplementary table 3, http://links.lww.com/MD/E366).

**Figure 1:**
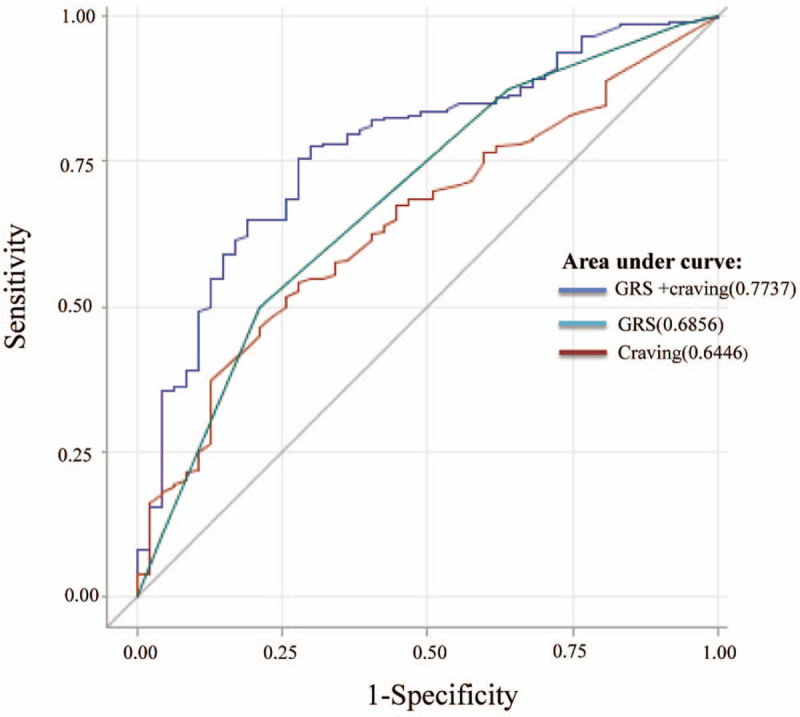
Receiver operating characteristic curves and comparison of areas under the ROC curves for the GRS and craving were used to predict maximum dose of MMT. GRS = genetic risk score, MMT = methadone maintenance treatment.

The prediction accuracy of the maximum MMT dose in the combined dataset is presented in Table 5. The diagnostic ability for the GRS and craving was measured using AUROC in the prediction models. The AUROCs of GRS and craving score were 0.69 (95% CI: 0.61–0.77; *P* < .0001) and 0.65 (95% CI: 0.56–0.73; *P* = .0002), respectively. The AUROC of the combined GRS and craving score was a significant predictor for maximum MMT dose, with 75% sensitivity and 60% specificity (Table 5; Fig. 1).

**Table 5:**
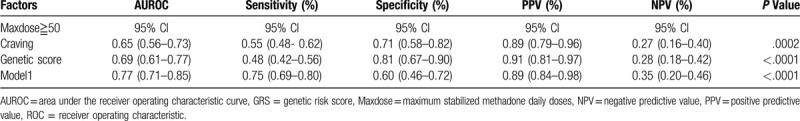
ROC curves for the GRS and craving were used to predict maximum stabilized methadone daily doses.

## 4 Discussion

The major findings of our study were as follows:

(1) Four susceptibility SNPs are associated with maximum stabilized methadone daily dose, independent of craving scores.
(2) For the risk prediction models, combined GRS and craving scores predict maximum stabilized methadone daily dose. Furthermore, we developed risk prediction models to identify individuals with high risk of heroin addiction and effective MMT doses.

We observed that rs806368 in *CNR1*, rs1386493 in *TPH2*, rs16974799 in *CYP2B6*, and rs2229205 in *OPRL1* were significantly associated with maximum MMT dose. Methadone has been studied most intensively in heroin addiction pharmacogenetics research.^[12]^ Several *ABCB1, CYP2B6,* and *OPRD1* variants have been associated with methadone plasma levels in candidate gene studies.^[12,13]^ In addition, 2 genome wide association studies (GWAS) identified significant associations of *CYP2B6* and *OPRM1* with methadone dose.^[14,15]^ Because of the chemical structure of methadone, the isozymes CYP2C19 and CYP2B6 metabolize the R-enantiomer^[16]^ and S-enantiomer^[17,18]^ of methadone in the liver, respectively. Genetics can play a role in methadone metabolism and the resulting plasma concentrations. These variants can serve as surrogate markers for MMT response and efficacy.

To the best of our knowledge, the present study is the first to report the genetic association of *CNR1* and *TPH2* with maximum MMT dose. *CNR1* on chromosome 6q14–15 encodes CB1, which likely modulates the release of serotonin, norepinephrine, and dopamine, disturbances of which are well documented in depression^[19]^ and addictive disorders.^[20]^*CNR1* SNPs are associated with nicotine,^[21]^ marijuana,^[22]^ alcohol,^[23]^ cocaine,^[24]^ and polysubstance use disorder.^[25]^ Moreover, we found that rs806368 in CNR1 is associated with maximum MMT dose and heroin addiction risk—consistent with the results of a study on CNR1 and its influence on lifetime major depressive disorder and suicidal behavior in a population of opiate-dependent outpatients remitted under stable methadone treatment.^[26]^

We also observed that patients with specific genotypes of *TPH2* are associated with maximum stabilized methadone daily dose. TPH2 activation may be considered a new prospective in the treatment of neuropsychiatric diseases related to brain 5-HT levels.^[27]^ TPH2 *gene* variants are related to the efficacy of disulfiram treatment for cocaine addiction.^[28]^ Because cocaine's mechanism of action involves blocking dopamine, norepinephrine, and serotonin transporters,^[29]^ we also evaluated the role of a functional variant in TPH2. We observed that patients with the CC genotype of TPH2 exhibited more favorable responses to methadone than did those patients without this genotype. This TPH2 variant has also been associated with heroin addiction^[30]^ and quality of life in the MMT population.^[31]^

We integrated genetic variations of known pharmacokinetic and pharmacodynamic genes as well as additional genes and craving scores to predict maximum MMT dosage. The genetic risk, calculated using the individual common genetic variants, was modest; their combination resulted in a large effect on maximum MMT dosage. However, GRS with higher frequencies were rare, suggesting that variants with large effect sizes might affect a small proportion of the population. Our genetic model was associated with higher methadone dose, suggesting that combining the GRS and craving score may help identify an adequate MMT dosage for individuals. The prediction model may be useful in defining individual MMT dose, to suggest initiation of methadone treatment, and at the start of treatment to predict the optimal dose for a particular patient. The strengths of our study include comprehensive addiction scale information and the utilization of multivariate logistic regression analysis to assess the combinatory effect of genetic polymorphisms on maximum MMT dosage.

Several limitations of this study must also be acknowledged. First, our sample size was small than that applied in previous related case–control studies. We performed a QTL association to identify genetic variants for MMT dosage. Our study did not include a control group; therefore, we suggest that larger-scale studies should be conducted to confirm our prediction model in practice without a genetic risk population.

The AUROCs for GRS and craving score were 0.69 and 0.65, respectively, but their sensitivity and specificity were low. Our study had only limited phenotypic and genotypic information. Moreover, the candidate gene approach allows the scanning of a limited number of SNPs. The identification of more genes associated with MMT dosage is imperative. This should include candidate genes identified in GWAS^[14,15,32,33]^ and variants underlying heroin addiction to increase accuracy in future studies.

Although the variants identified in this study produce relative risk for the response to MMT dosage, they may uncover potential mechanisms of addictive behavior. Future studies with patients undergoing methadone treatment could investigate variability in treatment response under the influence of environmental factors (eg, other pharmacological treatments, toxic exposure, harmful or safe physical and psychological environment, and lifestyle) and acting through epigenetic modifications and gene regulation.

## 5 Conclusion

Four susceptibility SNPs are associated with maximum MMT dosage.

(1) For the risk prediction models, combining GRS and craving score may be useful in the evaluation of individual MMT dose requirements at the start of treatment.
(2) Optimal dose prediction can allow clinicians to tailor MMT to each patient's needs.rmed consent;
(3) within normal EKG;
(4) not using concurrent medications which may affect methadone metabolism. The following clinical information was recorded for each patient: gender, weight (kg), height (cm), liver function, comorbidities and the daily dose of methadone.

### 5.1 HUC questionnaire

This study designed a 14-item questionnaires, heroin using and craving (HUC) score to evaluate the behaviours, for heroin abusers received methadone maintenance therapy. Each question scores 0 to 4. The questionnaire is divided into 2 parts: part I (HUC 1–HUC 6) is to assess the urge for heroin and whether one can shift the attention from heroin. The part I sum score is calculated range from 0 to 24. The part II (HUC 7–HUC 14) is to investigate the daily or weekly frequencies of heroin usage, daily life disturbance, anxiety emotion and the ability to overcome heroin use. The part II sum calculated score ranges from 0 to 32. The higher the score, the more severe the cravings for heroin use. The score of each item would be calculated according to the manual.

### 5.2 Candidate variants selection and genotyping

This study focused on 13 genes encoding components of the ( *OPRK1, OPRL1, PDYN* and CNR1), the dopaminergic and serotonin (*COMT*, TPH1 and TPH2) and Methadone-metabolizing enzymes (*CYP1A2, CYP2B6 and CYP2C1*), as well as the Cognitive function (*GRIN3A, GRIN3B and GRM6*). SNPs with minor allele frequency greater than 0.05 were selected based on previous studies and SNP databases of the National Center for Biotechnology Information CBH (Han Chinese in Beijing, China) population data. A total of 44 SNPs were genotyping at the National Center for Genome Medicine, Taiwan by the Sequenom iPLEX matrix-assisted laser desorption/ionization time-of-flight mass-spectrometry technology. DNA was extracted from 3 to 10 mL of whole blood by using the QIAamp DNA Blood mini Kit (Qiagen, Valencia, CA) according to the manufacturer's protocol. Duplicates samples were randomly selected for quality control and the concordance rate was >0.99 for all SNPs assayed.

We calculated an SNP risk score based on estimates of the OR per allele and risk allele frequencies (p) assuming independence of additive risks (22). A score of 1 was given to each T allele of *CNR1* - rs806368 and each C allele of *TPH2*- rs1386493, C allele of *CYP2B6* - rs16974799 and each C allele of *OPRL1* -rs2229205. The overall GRS was then calculated by the SNP risk alleles.

### 5.3 Statistical analysis

Each SNP genotype frequency distribution was examined for Hardy-Weinberg equilibrium by using the chi-square 1-degree of freedom goodness-of-fit test. Demographic characteristics and clinical parameters were evaluated with a chi-squared contingency table for categorical variables and t test for continuous variables between the gender groups. We performed QTL mapping for max methadone maintenance dose. The general linear model was used for associating max methadone maintenance dose with genotype data, making adjustments for sex, age, and BMI. The ranges of GRSs were from 0 to 4. A multinomial logistic regression model including GRS and craving interaction term adjusted for covariates was applied to examine in an association between max methadone maintenance dose and genetic variants. We employed AUROC curves to compare the accuracy of maximum dose of MMT. Sensitivity, specificity, and area under the receiver operating characteristic curve (AUROC) were used to evaluate the performance of the models. The optimal cutoff risk score threshold was identified at which both sensitivity and specificity were maximized (sensitivity + specificity). In addition, AUROC loss by stepwise selection was also performed to investigate important and determining factors of the model.

## 6 Results

### 6.1 Characteristics of subjects in the CMUH

No significant differences were observed between male and female groups with respect to heroin using and craving score, maximum dose, heroin use duration, education level and marital status. Significant differences were noted in the mean age, BMI and heroin onset age of the subjects (Table 1).

A total of 40 SNPs in 10 genes were not associated with maximum dose of MMT (supplementary table 1, http://links.lww.com/MD/E366). We noticed that 4 SNPs, rs806368 in *CNR1* gene, s1386493 in *TPH2* gene, s16974799 in the *CYP2B6* gene, and rs2229205 in the *OPRL1*, were significantly associated with maximum dose of MMT (*P* < .05; Table 2)

We constructed GRS by using 4 significant SNPs in the Table 3. The univariate associations of GRS and associated with maximum dose of MMT are shown in a dose-response way (Table 3). The GRS was associated with maximum dose of MMT, after adjustment for age, and gender and BMI, the GRS remained independently associated with t maximum dose of MMT.

### 6.2 Selection of predictive variables for maximum dose of MMT.

Based on the results of Table 3, we conducted stepwise model analysis to select the significant factors for the best fit to prediction model of maximum dose of MMT. The results were shown in the Table 4. CNR1 TPH2, CYP2B6, OPRL1 and craving were included in the best fit model (*P*-value was set < .05).

The prediction accuracy of the maximum dose of MMT is presented in Table 5. The diagnostic ability for GRS and craving was measured as AUROC in the prediction models. The AUROC for GRS was 0.69(95% CI: 0.61–0.77; *P* < .0001). The AUROC for craving were 0.65(95% CI: 0.56–0.73; *P* = .0002). The AUROC of the combined genetic and craving score were significant predictors for maximum dose of MMT with 46% of sensitivity and 85% of specificity for maximum dose of MMT, respectively (Table 5; Fig. 1).

## 7 Discussion

The major findings of our study are:

(1) Four susceptibility SNPs was associated with maximum stabilized methadone daily doses independent of craving scores
(2) For the risk prediction models, combined GRS and *f* craving scores was predicting maximum stabilized methadone daily doses.

Furthermore, we showed risk prediction models to identify individuals with high risk of heroin addiction and effective doses of MMT. We found that 4 SNPs, rs806368 in *CNR1* gene, rs1386493 in *TPH2* gene, rs16974799 in the *CYP2B6* gene, and rs2229205 in the *OPRL1*, were significantly associated with maximum dose of MMT. Methadone has been studied most intensively in heroin addiction pharmacogenetics research.^[12]^ Several variants in *ABCB1 CYP2B6 OPRD*1 genes were indentified association with methadone plasma levels in the candidate gene study.^[12,13]^ In addition, 2 GWAS identified *CYP2B6* and *OPRM1* genes significant association with methadone dose.^[14,15]^ Because of chemical structure of methadone, the CYP2C19 isozyme metabolizes methadone R-enantiomer^[16]^ and CYP2B6 isozyme metabolizes methadone S-enantiomer^[17,18]^ in the liver. The plasma concentration of methadone can be quantifying methadone metabolism via genetic effects. These variants can serve as a surrogate marker for the treatment responses and efficacy of MMT.

To the best of our knowledge, the present study is the first study to report the genetic association between *CNR1* and *TPH2* and maximum dose of MMT. The cannabinoid receptor 1 gene (*CNR1*) on chromosome 6q14- 15 encoded CB1 protein that is likely to modulate the release of serotonin, norepinephrine and dopamine systems, disturbances of which are well documented in depression^[19]^and addictive disorders.^[20]^ Several studies reported that SNPs of *CNR1*were associated with nicotine,^[21]^ marijuana,^[22]^ alcohol,^[23]^ cocaine,^[24]^ and poly-substance use disorder.^[25]^ We demonstrate association between polymorphisms (rs806368) in the CNR1 gene and maximum dose of MMT and the risk of heroin addiction. This finding is consistent with previous studies of CNR1 and its influence on lifetime major depressive disorder and suicidal behavior in a population of opiate-dependent outpatients remitted under stable methadone treatment.^[26]^

We have found that those patients with specific genotypes of tryptophan hydroxylase 2 genes are associated with maximum stabilized methadone daily doses. TPH2 activation may be regarded as a new prospective for neuropsychiatric diseases related to brain 5-HT levels.^[27]^ The variants inTPH2 *gene* was related to the efficacy of disulfiram treatment for cocaine addiction.^[28]^ Since cocaine's mechanism of action is through blockade of dopamine, norepinephrine and serotonin transporters,^[29]^ we also evaluated the role of a functional variant in the TPH2 gene. We found that patients with CC genotype ofTPH2 responded to methadone better than those patients without this genotype. The variant in TPH2 also has been found to be associated with heroin addiction^[30]^ and quality of life in methadone maintenance therapy population^[31]^

We integrated genetic variation at known pharmacokinetic and pharmacodynamic genes, as well as additional genes and craving score to apply prediction for maximum dosage of MMT. The genetic risk calculated by the individual common genetic variants was modest; their combination resulted in a large effect on maximum dosage of MMT. However, the frequencies of higher GRS were rare, suggesting that variants with large effect sizes will impact a small proportion of the population. Our genetic model was associated with higher methadone dose, suggesting that combine GRS and craving score may help identify an adequate dosage of MMT in the setting of addiction treatment for individual. The efficacy of prediction model may be useful in individual MMT dose requirements to suggest initiation of methadone treatment and at the start of treatment to predict the optimal dose for a particular patient. The strengths of our study include comprehensive addiction scale information and utilized multivariate logistic regression analysis to assess the combinatory effect of genetic polymorphisms on the maximum dosage of MMT.

Several limitations must also be acknowledged. First, the sample size is small compared with case/control cross many different studies. We performed a QTL association to identify genetic variants for MMT dosage. Without normal control in this study, we have suggested that larger-scale studies are warranted to further confirm this prediction model used practically without genetic risk population.

The AUROCC for GRS and craving score were 0.69 and 0.65 respectively. The sensitivity and specificity for GRS and craving were relatively lower. Our study had only limited phenotypic and genotypic information. The candidate gene approach allows a scan of a limited number of SNPs. We needed to identify more genes associated with dosage of MMT, include candidate genes identified by GWAS^[14,15,32,33]^ and variants underlying heroin addiction to increase accuracy in the future study.

Although the variants identified in this study are suggested to produce relative risk for the response to dosage of MMT, they may uncover potential mechanisms of addictive behavior. In particular, future recruitment of additional methadone- treated patients will be needed in order to study variability in treatment response under the influence of environmental factors (ie, other pharmacological treatments, toxic exposure, harmful or safe physical and psychological environment, lifestyle and so on) and acting through epigenetic modifications and gene regulation.

## 8 Conclusion

In summary, our study provided evidence that 4 susceptibility SNPs was associated with maximum dosage of MMT. For the risk prediction models, we combined GRS and craving score may be useful in evaluation of individual MMT dose requirements at the start of treatment. Predicting optimal dose for each individual can allow the clinician to provide carefully the dose to each individual patient's needs.

## Acknowledgments

We thank the National Center for Genome Medicine for the technical support.

## Author contributions

CLH and RYW were responsible for the study concept and design. HWC and WCH conducted data analysis and interpretation of findings. CLH and RYW interpreted results of genotyping and clinical data. HWC, WCH and RYW drafted the manuscript. All authors critically reviewed content and approved the final version for publication.

## Supplementary Material

SUPPLEMENTARY MATERIAL
