# Supplementary material for: Precision therapeutic opioid dosing implications from genetic biomarkers and craving score
Source: Medicine (Baltimore). 2020 May 29;99(22):e20429. doi: 10.1097/MD.0000000000020429 (PMC12245365; doi:10.1097/MD.0000000000020429)
Supplement: SUPPLEMENTARY MATERIAL [file medi-99-e20429-s001.doc]

**Supplementary table 1**. **Comparing characteristics of training set and test set.**

|  | **Training set (n=229)** | **Test set(n=97)** |  |
| --- | --- | --- | --- |
| **Variables** | **Mean(SD)** | **Mean(SD)** | **p-value** |
| Age | 42.3(7.5) | 41.6(7.0) | 0.4514 |
| BMI | 22.7(2.8) | 22.8(2.8) | 0.6914 |
| Heroin using and craving score | 27.1(9.4) | 27.9(11.8) | 0.5011 |
| Urge for heroin | 10.5(4.5) | 10.9(5.1) | 0.4213 |
| Ability to overcome heroin use | 16.7(5.8) | 17.3(7.0) | 0.6269 |
| Maxdose | 73.7(29.9) | 75.0(31.4) | 0.7335 |
| Heroin onset age( year) | 25.4(7.4) | 24.4(7.2) | 0.2573 |
| Heroin use duration(year) | 8.8(6.2) | 8.0(5.2) | 0.5023 |

**Supplementary table 2**. **Associations between SNPs and maximum stabilized methadone daily doses** **in 15 candidate genes in all cases.**

| **Gene** | **SNP** | **Allele** | **chromosome** | **position** | **p-vlaue** |
| --- | --- | --- | --- | --- | --- |
| GRM6 | rs17078853 | G>T | 5 | 178981601 | 0.8676 |
| GRM6 | rs2071247 | A>G | 5 | 178983150 | 0.3281 |
| GRM6 | rs17078877 | G>A | 5 | 178983212 | 0.8363 |
| GRM6 | rs11746675 | C>T | 5 | 178986946 | 0.8866 |
| GRM6 | rs2067011 | C>T | 5 | 178988936 | 0.9473 |
| CNR1 | rs806368 | C>T | 6 | 88140381 | <.0001 |
| CNR1 | rs806380 | A>G | 6 | 88154934 | 0.3185 |
| OPRK1 | rs6473799 | G>A | 8 | 53240563 | 0.922 |
| GRIN3A | rs7030238 | C>A | 9 | 101570213 | 0.4177 |
| GRIN3A | rs1983812 | G>A | 9 | 101570761 | 0.6148 |
| GRIN3A | rs942142 | C>A | 9 | 101670591 | 0.6843 |
| GRIN3A | rs10512285 | A>G | 9 | 101670752 | 0.3318 |
| GRIN3A | rs3983721 | C>T | 9 | 101733129 | 0.2107 |
| CYP2C1 | rs6583954 | T>C | 10 | 94774506 | 0.7569 |
| CYP2C1 | rs11528090 | G>T | 10 | 94838682 | 0.1917 |
| TPH1 | rs211105 | G>T | 11 | 18033757 | 0.8532 |
| TPH1 | rs623580 | T>A | 11 | 18042430 | 0.0702 |
| TPH2 | rs2129575 | G>T | 12 | 71946293 | 0.6498 |
| TPH2 | rs1386493 | C>T | 12 | 71961399 | 0.0277 |
| TPH2 | rs2171363 | T>C | 12 | 71966484 | 0.1591 |
| TPH2 | rs7305115 | A>G | 12 | 71979082 | 0.1196 |
| TPH2 | rs10506645 | T>C | 12 | 71991720 | 0.6324 |
| TPH2 | rs4760820 | C>G | 12 | 72003216 | 0.7918 |
| TPH2 | rs9325202 | A>G | 12 | 72013697 | 0.081 |
| TPH2 | rs1487275 | G>T | 12 | 72016512 | 0.0638 |
| TPH2 | rs1487275 | G>T | 12 | 72016512 | 0.0638 |
| CYP1A2 | rs4646425 | C>T | 15 | 74750940 | 0.4953 |
| GRIN3B | rs2240158 | T>C | 19 | 1005231 | 0.1234 |
| GRIN3B | rs2285906 | G>A | 19 | 1008684 | 0.8984 |
| GRIN3B | rs2285907 | C>G | 19 | 1008880 | 0.9924 |
| CYP2B6 | rs3760657 | A>G | 19 | 40989528 | 0.6339 |
| CYP2B6 | rs16974799 | C>T | 19 | 40998172 | 0.0035 |
| PDYN | rs910080 | A>G | 20 | 1979580 | 0.636 |
| PDYN | rs2235751 | G>A | 20 | 1989288 | 0.9561 |
| PDYN | rs1997794 | T>C | 20 | 1994212 | 0.2069 |
| OPRL1 | rs6010717 | C>G | 20 | 64083430 | 0.6041 |
| OPRL1 | rs7271530 | C>T | 20 | 64096562 | 0.7233 |
| OPRL1 | rs2229205 | T>C | 20 | 64098078 | 0.0433 |
| COMT | rs2020917 | T>C | 22 | 19941361 | 0.4437 |
| COMT | rs933271 | C>T | 22 | 19943884 | 0.5294 |
| COMT | rs174675 | C>T | 22 | 19946528 | 0.5383 |
| COMT | rs5993882 | G>T | 22 | 19950010 | 0.2717 |
| COMT | rs174699 | C>T | 22 | 19966935 | 0.4838 |
| COMT | rs174699 | C>T | 22 | 19966935 | 0.4838 |

**Supplementary table 3**. **The prediction model for maximum stabilized methadone daily doses was evaluated by the area under the ROC curve in both training and testing sets.**

| **Data set^＊^** | **Factors** | **AUROC** | **Sensitivity (%)** | **Specificity (%)** |
| --- | --- | --- | --- | --- |
| **Training set** | **Craving** | 0.63(0.53-0.73) | 0.54(0.45- 0.62) | 0.69(0.53-0.82) |
| **Training set** | **Genetic score** | 0.68(0.58-0.78) | 0.51(0.41-0.59) | 0.76(0.61-0.90) |
| **Training set** | **Model** | 0.75(0.67-0.84) | 0.76(0.68-0.83) | 0.54(0.37-0.68) |
| **Test set** | **Caving** | 0.67(0.51-0.83) | 0.54(0.41- 0.66) | 0.82(0.69-0.93) |
| **Test set** | **Genetic score** | 0.70(0.57-0.83) | 0.42(0.33-0.51) | 0.93(0.80-0.99) |
| **Test set** | **Model** | 0.81(0.70-0.92) | 0.79(0.71-0.87) | 0.67(0.51-0.83) |

**^＊^**Training and test sets were split 70% (*n* = 229) and30% (*n* =97), respectively.
